# Resistance Exercise and Whey Protein Supplementation Reduce Mechanical Allodynia and Spinal Microglia Activation After Acute Muscle Trauma in Rats

**DOI:** 10.3389/fphar.2021.726423

**Published:** 2021-11-11

**Authors:** Gusthavo Rodrigues, Thamyris Moraes, Lívia Elisei, Iago Malta, Rafaela dos Santos, Rômulo Novaes, Pablo Lollo, Giovane Galdino

**Affiliations:** ^1^ Laboratory of Experimental Physical Therapy, Institute of Motricity Sciences, Federal University of Alfenas, Alfenas, Brazil; ^2^ Federal Institute of Education, Science and Technology of South of Minas Gerais, Advanced Campus Carmo de Minas, Carmo de Minas, Brazil; ^3^ School of Physical Education, Federal University of Grande Dourados, Dourados, Brazil

**Keywords:** resistance exercise, whey protein, allodynia, antinociception, glial cells

## Abstract

Muscle injury caused by direct trauma to the skeletal muscle is among the main musculoskeletal disorders. Non-pharmacological treatments have been effective in controlling muscle injury–induced pain; however, there are just a few studies in the literature investigating this response. Thus, the present study aimed to evaluate the effect of a resistance exercise training protocol combined or not with whey protein supplementation on mechanical allodynia induced by muscle injury. In addition, we also investigated the involvement of spinal glial cells in this process. For this purpose, male Wistar rats underwent a muscle injury model induced by direct trauma to the gastrocnemius muscle. Mechanical allodynia was measured by a digital von Frey algesimeter test. To evaluate the effect of exercise and/or supplementation on mechanical allodynia, the animals practiced exercises three times a week for 14 days and received supplementation daily for 14 days, respectively. Moreover, the effect of both the participation of spinal glial cells in the muscle injury and the resistance exercise training and/or whey protein supplementation on these cells was also investigated by the Western blot assay. The results demonstrated that resistance exercise training and whey protein supplementation, combined or alone, reduced mechanical allodynia. These treatments also reduced the number of interstitial cells and pro-inflammatory cytokine IL-6 levels in the injured muscle. It was also found that spinal microglia and astrocytes are involved in muscle injury, and that resistance exercise training combined with whey protein supplementation inhibits spinal microglia activation. The results suggest that both resistance exercise training and whey protein supplementation may be effective non-pharmacological treatments to control pain in the muscle after injury induced by acute trauma.

## Introduction

Skeletal muscle injuries are common during sports practice and comprise 10–55% of all injuries (Delos et al., 2013). These injuries are often accompanied by severe and lasting pain, which can impact the functionality, emotional health, and the quality of life of the affected individuals (Gigante et al., 2014). Furthermore, the muscle injury recovery process depends on relatively well-established phases, such as degeneration, inflammation, and regeneration (Laumonier and Menetrey, 2016).

Inflammation associated with muscle regeneration is complex since it relies on the balance between pro- and anti-inflammatory factors (Laumonier and Menetrey, 2016). Therefore, the inflammatory phase is an important period for the application of therapies aiming at its resolution (Alessandri et al., 2013). During muscle inflammation, there are classical signs and symptoms, including loss of function, hyperthermia, edema, and erythema (Gibbins, 2018), which may cause discomfort or even pain to the individual.

According to the International Association for the Study of Pain (IASP), pain is an unpleasant sensory and emotional experience associated with actual or potential tissue damage and described in terms of such damage (Treede, 2018). Thus, during inflammation following muscle injury, inflammatory mediators are released, peripherally sensitizing pain receptors (referred to as nociceptors), and consequently initiating the painful response (Gibbins, 2018; Treede, 2018). In addition to the peripheral mechanisms, central mechanisms also contribute to pain modulation. Among them, spinal glial cells stand out.

Glial cells are activated in the central nervous system after persistent nociceptive stimuli in the periphery (Ji et al., 2016). This activation leads to the sensitization of the nociceptive pathway by producing and releasing several pro-inflammatory signaling molecules, which in turn increase the excitability of nociceptive neurons and the overall perception of pain—a response known as “central sensitization” (Ji et al., 2016). Glia mainly comprises three types of cells: astrocytes, microglia, and oligodendrocytes, with the first two being the most frequently associated with nociceptive modulation (Ji et al., 2013). However, a few studies have investigated the participation of glia in muscle injury–induced pain.

Some studies have reported the efficiency of non-pharmacological treatment strategies against muscle injuries, among which resistance exercise training has been proven beneficial to control pain (Bertolini et al., 2012; Galdino et al., 2014; Jones, 2015). In addition to resistance exercise training, the ingestion of supplements, such as whey protein, may attenuate muscle microinjuries, pain, and local macrophage infiltration caused by some muscular stress resulting from exercise and cryolesion (Pereira et al., 2014; Kato et al., 2016; Qin et al., 2019). However, to the best of our knowledge, no other study has investigated the effect of the interaction between resistance exercise training and ingestion of whey protein on mechanical allodynia, as well as on the participation of spinal glia cells during muscle injury. Thus, the present study aimed to evaluate the effect of resistance exercise training and whey protein supplementation on mechanical allodynia induced by a muscle injury model in rats. Moreover, this study explored the contribution of spinal glial cells to muscle injury, as well as the influence of such non-pharmacological treatments on this process.

## Materials and Methods

### Animals

The experiments were conducted with male Wistar rats (220–250 g) obtained from the vivarium of the Federal University of Alfenas-UNIFAL (Alfenas, Minas Gerais, Brazil). All animals were housed under standard conditions at a temperature of 23 ± 1°C and humidity of approximately 50% with a 12:12 h light/dark cycle. The animals were acclimatized to the laboratory for 1 h before the evaluations, which were carried out between 8:00 a.m. and 17:00 p.m. with free availability of water and feed. All procedures were approved by the Local Ethics Committee on Animal Experimentation (protocol number 651/2015) and are in accordance with the recommendations for the evaluation of experimental pain in animals (Zimmermann, 1983).

### Experimental Protocol

For the experiments described below, the animals were randomly divided into the following groups (*n* = 5–6): naive, composed of rats not subjected to the muscle injury model; MI, comprising rats subjected to the muscle injury model; MI + RET, formed by rats subjected to the muscle injury model and the resistance exercise training; MI + WP, composed of rats subjected to the muscle injury model and that received whey protein supplementation; and finally MI + RET + WP, comprising rats subjected to the muscle injury model, followed by resistance exercise training and whey protein supplementation. In addition, in some experimental groups, we also evaluated the effect of whey protein supplementation vehicle or resistance exercise training in naïve rats by comparing them to rats not subjected to the muscle injury model, and that received whey protein supplementation vehicle (saline); rats subjected to the muscle injury model and that received saline; rats not subjected to the muscle injury model and that received whey protein supplementation; rats not subjected to the muscle injury model and that performed resistance exercise training; and last, rats not subjected to the muscle injury model that received a combination of saline and resistance exercise training.

**FIGURE 1:**
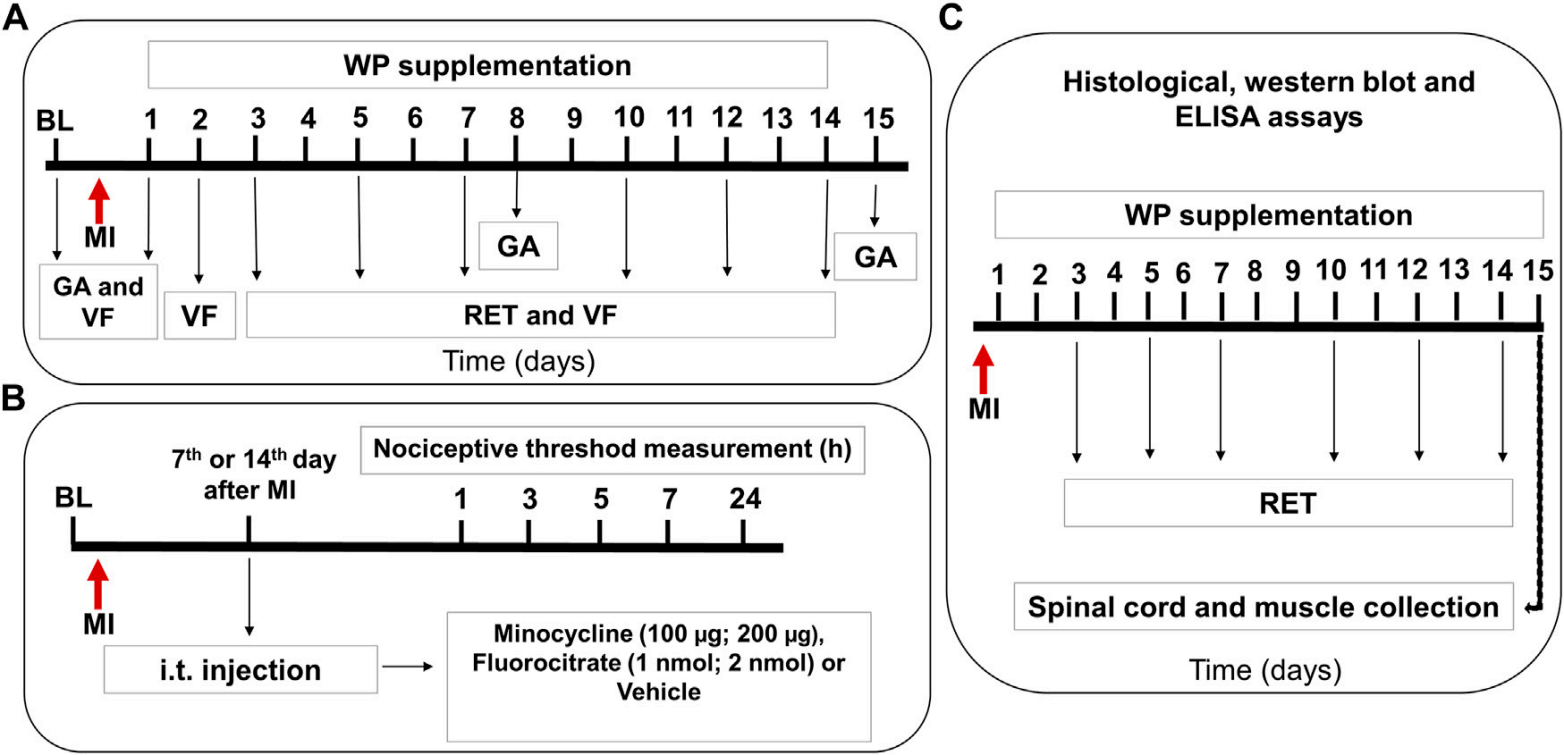
Representative scheme of experimental protocols for the evaluation of muscle injury–induced nociception. Panel **(A)** shows that after evaluation of the baseline (BL) for gait (GA) or nociceptive threshold by the digital von Frey test (VF), the muscle injury (MI) was induced. After that, the rats were subjected to daily supplementation with whey protein (WP) and/or resistance exercise training (RET, 3 days a week for 2 weeks). A new measurement of the nociceptive threshold was performed after 1, 2, 3, 5, 7, 10, 12, and 14 days, and a new GA analysis was performed after 1, 8, and 15 days. Panel **(B)** shows the evaluation of involvement of the spinal microglia and astrocytes in the muscle injury–induced nociception. After BL evaluation, the animals were subjected to MI, and after 7- and 14-days of minocycline, fluorocitrate or vehicle was intrathecally injected. Panel **(C)** shows that after MI, the rats underwent WP supplementation and/or RET, and after 15 days, they were euthanized to collect the spinal cord and muscles that were later used for histological and Western blot analysis.

**FIGURE 2:**
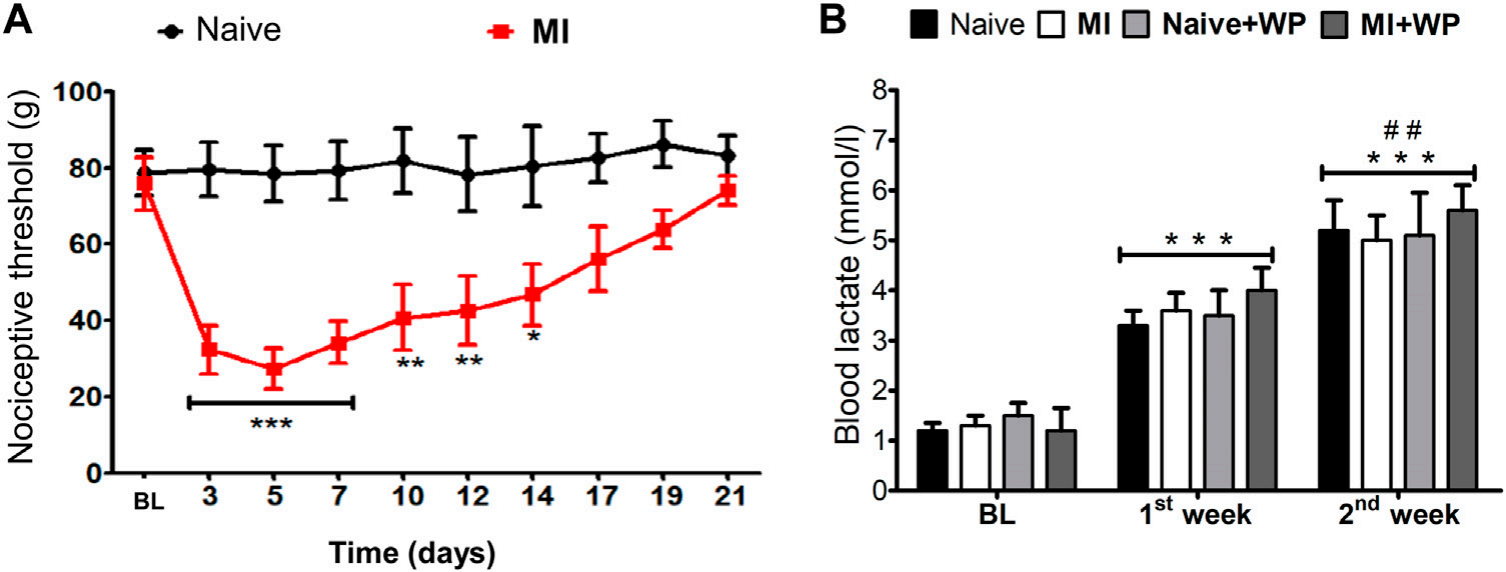
Effect of the muscle injury on the nociceptive threshold and of resistance exercise on the blood lactate levels. First, the baseline nociceptive threshold (BL) of each animal was measured and then the muscle injury (MI) was induced **(A)**. After that, and every 2 days from the 3rd to the 21st day, a new measurement of the nociceptive threshold was performed. **(B)** Blood lactate levels were given dosage before (BL) and after the 1st and 2nd weeks of resistance exercise training (RET) and/or whey protein (WP) supplementation in rats with MI or naïve (without MI). Data are expressed as the mean ± S.E.M. of six animals per group. In **(A)**, *p < 0.05, **p < 0.01, and ***p < 0.001 indicate statistical significance compared to the naïve group; in **(B)**, ***p < 0.001 indicates statistical significance compared to baseline levels (BL); ^##^ *p* < 0.01 indicates statistical significance between the 1st and 2nd weeks. **(A)** Two-way ANOVA followed by the Bonferroni *post hoc* test; **(B)** one-way ANOVA was followed by the Bonferroni *post hoc* test.

Thus, a first experiment was carried out with animals belonging to the groups described above in order to evaluate the effect of resistance exercise training and/or whey protein supplementation on the nociceptive threshold and the gait (Figure 1A). For this purpose, the baseline assessment for the gait or the nociceptive threshold was initially measured, followed by muscle injury induction. Thereafter, resistance exercise training was performed on days 3, 5, 7, 10, 12, and 14, whereas whey protein supplementation was administered from the 1st to the 14th day. In addition, new measurements of nociceptive threshold were performed on 1, 2, 3, 5, 7, 10, 12, and 14 days after muscle injury, and new gait analyses were performed after 1, 8, and 15 days (Figure 1A). In order to verify whether the trauma-induced muscle injury model would alter the nociceptive threshold, this was evaluated in the first experiment until the 21st day. However, after the 14th day, it was not possible to verify mechanical allodynia, which made us choose this period for the next experiment (Figure 2A).

A second experiment involving other groups of animals was carried out to investigate the involvement of spinal glial cells in mechanical allodynia after trauma-induced muscle injury (Figure 1B). Thus, after the baseline measurement, specific inhibitors for microglia and astrocytes (minocycline and fluorocitrate, respectively) were intrathecally administered on days 7 and 14 after injury, and new measurements of nociceptive threshold were performed after 1, 2, 3, 7, 24, and 48 h of injection (Figure 1B).

Another experiment used new animals referring to the groups described above for blood lactate dosage, histopathological analysis, and the Western blot assay (Figure 1C). Blood lactate levels were measured before (baseline) and after the first and second weeks of exercise and/or whey protein supplementation in injured or naïve (without muscle injury) rats (Figure 1C). Furthermore, at the end of the treatments (resistance exercise training and/or whey protein supplementation), that is, on the 14th day, we collected the right gastrocnemius muscle and spinal cord of the animals, which were posteriorly used for histological analysis and Western blotting, respectively.

Finally, new groups of animals were used to perform the ELISA assay (Figure 1C). For this experiment, the right gastrocnemius muscle was collected on the 14th day after the treatments (resistance exercise training and/or whey protein supplementation).

### Drugs and Substances

In the present study, the following drugs were used: minocycline, a microglia inhibitor, purchased from Sigma-Aldrich (St. Louis, United States); fluorocitrate, an astrocyte inhibitor, purchased from Invivogen (San Diego, United States); and hydrolyzed whey protein (Alacen 895), obtained from NZMP (Santa Rosa, United States). All drugs and whey protein were diluted in sterile saline (0.9%).

### Intrathecal Injections

Prior to intrathecal (i.t.) injections, the rats were lightly anesthetized with isoflurane (2%) and O_2_ (2 L/min). Then, the lumbar region was shaved and cleaned, and the rats were positioned in the ventral decubitus to facilitate the palpation of the L5–L6 intervertebral spaces (Hylden and Wilcox, 1980). The i.t. injection in rats was administered in this intervertebral space using a 13 × 0.3-mm Hamilton needle (Hylden and Wilcox, 1980).

### Muscle Injury Model

The muscle injury model can be reproduced by acute trauma (Falcai et al., 2010). First, the rats were deeply anesthetized intraperitoneally with ketamine (12 mg/kg) and xylazine (1 mg/kg). Subsequently, the animals were positioned in a prone position, and the posterior surface of the hind limb was placed up by extending the knee and dorsiflexing the foot to 90°. Injury was produced by a metallic mass (2-cm base, 8-cm high, and 0.8-mm flat tip, 250 g) dropped from a plastic guide tube (60 cm in height and 2 cm in diameter) onto the posterior surface of the right gastrocnemius muscle.

### Whey Protein Supplementation

Whey protein supplementation was administered daily *via* the oral route by gavage (composition described in Table 1) for 2 weeks. Gavage was performed in a volume of approximately 1 ml/kg, at a concentration of 1 g/kg (Kerasioti et al., 2018).

**TABLE 1:** Composition of major amino acids in hydrolyzed whey protein Alacen 895.

Amino acids	Typical amount per 100g of protein (g)
Essential amino acids	
Isoleucine	6.2
Leucine	14.0
Lysine	11.1
Methionine	2.9
Phenylalanine	3.8
Threonine	5.0
Tryptophan	2.4
Valine	5.7
Non-essential amino acids	
Histidine	2.1
Alanine	5.9
Arginine	2.8
Aspartic acid	11.6
Cysteine	3.8
Glutamic acid	18.2
Glycine	1.8
Proline	4.6
Serine	3.9
Tyrosine	4.2

### Resistance Exercise Training Protocol

The resistance exercise training protocol involved a weight-lifting exercise model (Tamaki et al., 1992). This model is performed in an apparatus with a 35-cm wooden arm fastened at one end in the axis of the vertical gyration. In addition, an aluminum holder for rats was attached to the other end of the arm, which remained at an angle of 65°. Before starting the exercise, the rats wore a canvas jacket attached to the aluminum holder to keep them in an orthostatic position with the hind limbs flexed. An electrical stimulation (3 V, 0.3-s duration at 3-s intervals) was applied to the rat’s tail through a surface electrode. As a result, the rats extended their legs repeatedly, lifting the weight on the arm of the training apparatus. The animals were allowed to exercise for three sets of 10 repetitions, with a 120-s rest between each set. The resistance exercise training was performed three times a week for 2 weeks. The progressive load increase on each training day was based on the measurement of the maximum weight lifted (1 repetition maximum), which was performed before the training protocol and after each training day. Specifically, the percentage of 1 repetition maximum adopted for each day was as follows: 30, 40, and 50% for days 3, 5, and 7, respectively, and 60, 70, and 80% for days 10, 12, and 14, respectively.

### Measurement of the Blood Lactate Level

To assess whether resistance exercise training imposed a physical overload in the rats, blood lactate levels were measured. The measurement was performed at the end of each week of training through a digital lactate analyzer (Accutred Plus, Roche, Switzerland) by collecting blood in the caudal region of the animal. The first collection was performed on the first day after muscle injury, with the animals at rest and prior to the training protocol. Subsequently, blood was collected 3 min after the end of the third set of the last session of each resistance training week.

### Nociceptive Test

The mechanical nociceptive threshold was measured by a digital von Frey algesimeter (Ugo Basile, Italy). To this end, the rats were placed on an elevated wire mesh platform in individual glass compartments and allowed to acclimate for at least 30 min. Subsequently, a mechanical stimulus was applied to the middle of the right plantar surface, and the pressure value (in g) during paw withdrawal was recorded (Tena et al., 2012). The reported results represent the mean value of three consecutive tests applied at 3-min intervals.

### Gait Analysis

Gait analyses were performed according to a previous study (Varejão et al., 2002), which used joint amplitude and plantar contact area as the main evaluation parameters. The analyses were carried out using Kinovea software (Patreon, France).

### Microstructural Muscle Analysis

After the second week of treatment, all rats that underwent treatments, as well as the naïve group, were euthanized with an overdose of ketamine and xylazine. Afterward, the right gastrocnemius muscles were collected and dissected to expose the lesion area (pale red color, ventral muscle area), while the corresponding muscle area in control animals was also collected. Muscle fragments were th

en fixed by immersing in 10% formalin prepared in phosphate-buffered saline (PBS, 0.1M, and pH 7.2) for 24 h and embedded in histological glycol methacrylate resin (Leica Microsystems, Wetzlar, Germany). Then, six non-serial 3-μm-thick sections were obtained using a rotary microtome so that one in every 50 sections was collected to avoid analyzing the same histological area (Novaes et al., 2016). The sections were stained with hematoxylin and basic fuchsin for the histological analysis. In each histological section, digital images were captured from five random non-coincident histologic fields using a digital photographic camera (DsFi1, Nikon, Tokyo, Japan) coupled to an optical microscope (Eclipse 80i, Nikon, Tokyo, Japan) with a 40x objective lens and an image capture program (NIS-Elements, Nikon, Tokyo, Japan). A total of thirty histological fields of the gastrocnemius muscle were analyzed for each animal.

Muscle inflammation was analyzed by the stereological method previously described (Novaes et al., 2017). Considering muscle anisotropy (Novaes et al., 2018), vertical sections were used to reduce measurement bias. The inflammatory tissue damage was determined by comparing the interstitial cellularity in control and treated rats (Novaes et al., 2016; Novaes et al., 2017). The number density of all interstitial cells (QAic) was quantified in longitudinal sections according to the following formula: QAic = ∑QA/AT, where ∑QA is the number of interstitial cells in the test area applied at the tissue level (AT = 3.15 × 103 µm^2^) (Novaes et al., 2017). Stereological measurements were obtained from the Image-Pro Plus 4.5 software (Media Cybernetics, Rockville, MD, United States).

### Determination of Cytokine IL-6 in the Muscle

To evaluate the role of resistance exercise training and/or whey protein supplementation in pro-inflammatory cytokine interleukin 6 (IL-6) after trauma-induced muscle injury, the enzyme-linked immunosorbent assay (ELISA) was performed. To this end, the rats were deeply anesthetized with isoflurane (2%) and euthanized by decapitation. Then, the right gastrocnemius muscle was collected, and the IL-6 levels were determined using a commercially available ELISA kit. Subsequently, the samples were transferred to 1.5 ml tubes, placed on dry ice, resuspended in 1 ml of 0.1 M PBS, and homogenized using a tissue homogenizer. After washing, the samples or standards were incubated for quantification. After the addition of the detection antibody, the samples were incubated with avidin peroxidase solution. A chromogenic solution was then added, and the colorimetric reactions were analyzed at 405 nm using a microplate reader. Calculations were performed according to the standard curve for the determination of sample concentrations.

### Ionized Calcium-Binding Adaptor Molecule-1 and Glial Fibrillary Acidic Protein Levels

In order to evaluate the spinal microglia and astrocyte protein levels, a Western blot analysis was performed. For this purpose, the animals were euthanized by decapitation, and immediately afterward, the samples from the spinal cord (segments L4–L6) were removed carefully, homogenized in radioimmunoprecipitation assay (RIPA) buffer with a cocktail of protease inhibitors (Sigma-Aldrich, MO, United States), and stored in a freezer at −80°C. The samples were separated by centrifugation at 15,000 rpm for 10 min, and the protein concentration in lysate was measured by the Bradford method. Samples containing the protein mixtures in the Laemmli sodium dodecyl sulfate buffer were boiled and subjected to immunoblot analysis. The proteins were separated by 10–15% sodium dodecyl sulfate polyacrylamide gel electrophoresis (SDS–PAGE) and transferred onto nitrocellulose membranes with a semi-dry electrophoretic system (Bio-Rad, Hercules, CA). The membranes were washed twice in TBS containing 0.05% Tween 20, blocked with 5% nonfat milk for 2 h at room temperature, and then incubated overnight at 4°C with a rabbit anti-Iba1 (1:1,000, Wako, Japan) or a mouse anti-GFAP (1:250, Boster Biological Technology, United States) antibody. After three washes under the same conditions, the membranes were incubated for another 2 h at room temperature with anti-rabbit (1:2,000, Sigma-Aldrich, United States) and anti-mouse (1:3,000, Sigma-Aldrich, United States) secondary antibodies, respectively. After the incubation period, the membrane was washed again in TBS containing 0.05% Tween 20, and a commercial detection kit was applied for 3 min (ECL detection kit, Bio-Rad, Hercules, CA). Immunoblot analysis images were captured using a chemiluminescence imaging analyzer (Chemidoc, Bio-Rad, Hercules, CA). Subsequently, the membranes were stripped and incubated with anti–β-actin (1:5,000, A2228, Sigma-Aldrich, United States) for 2 h, washed in TBS, and incubated with their specific secondary antibody, and washed again. Further images were then captured using the device previously mentioned. The images of four membranes were quantified using Image Lab software (Bio-Rad, Hercules, CA), and the bands were quantified with the same area intensity, which were normalized to β-actin (used as internal control). Data were expressed as a fold change of the mean ± SEM in relation to the mean of controls.

### Statistical Analysis

The data are presented as mean ± standard error of mean (SEM). The Kolmogorov–Smirnov test was used to verify the normality of data. After that, the parametric data were analyzed by one-way ANOVA for biochemical assays or two-way ANOVA with repeated measures for behavioral experiments, followed by the Bonferroni *post hoc* test. Differences with a value of *p* < 0.05 were considered significant. The statistical analyses were performed using GraphPad Prism 5.0 software (GraphPad Software, La Jolla, CA).

## Results

### Allodynia Induced by the Trauma Muscle Injury Model

The results displayed in Figure 2A demonstrate a significant reduction of the nociceptive threshold in animals that underwent the muscle injury protocol from days 3 to 7 after the lesion [F(1,10) = 5.95; *p* < 0.001]. It can also be observed that this response lasted 14 days [F(1,10) = 5.95; *p* < 0.05] compared to the response in naïve animals.

### Physical Overload Induced by Resistance Exercise

After validating the trauma-induced muscle allodynia model, the next step of the study was to evaluate whether resistance exercise training would induce physical overload in the animals. We found that lactate levels increased at the end of the first and second weeks [F(3,20) = 13.42; *p* < 0.001] in animals with and without muscle injury (naïve) that received or not whey protein supplementation. Furthermore, after the second week, the lactate blood levels were higher than those in the first week [F(3,20) = 13.42; *p* < 0.01], indicating that the training carried out in this week (at 80% of 1 repetition maximum) induced a physical load greater than that in the first week (at 50% of 1 repetition maximum). Despite the increase in the lactate levels, there was no difference among the groups in these weeks. These data suggest that the resistance exercise training protocol efficiently generated a physical overload, a phenomenon that may result in muscle strength gain (Figure 2B).

### Reduction in Mechanical Allodynia Induced by Muscle Injury due to Resistance Exercise Training and Whey Protein Supplementation

**FIGURE 3:**
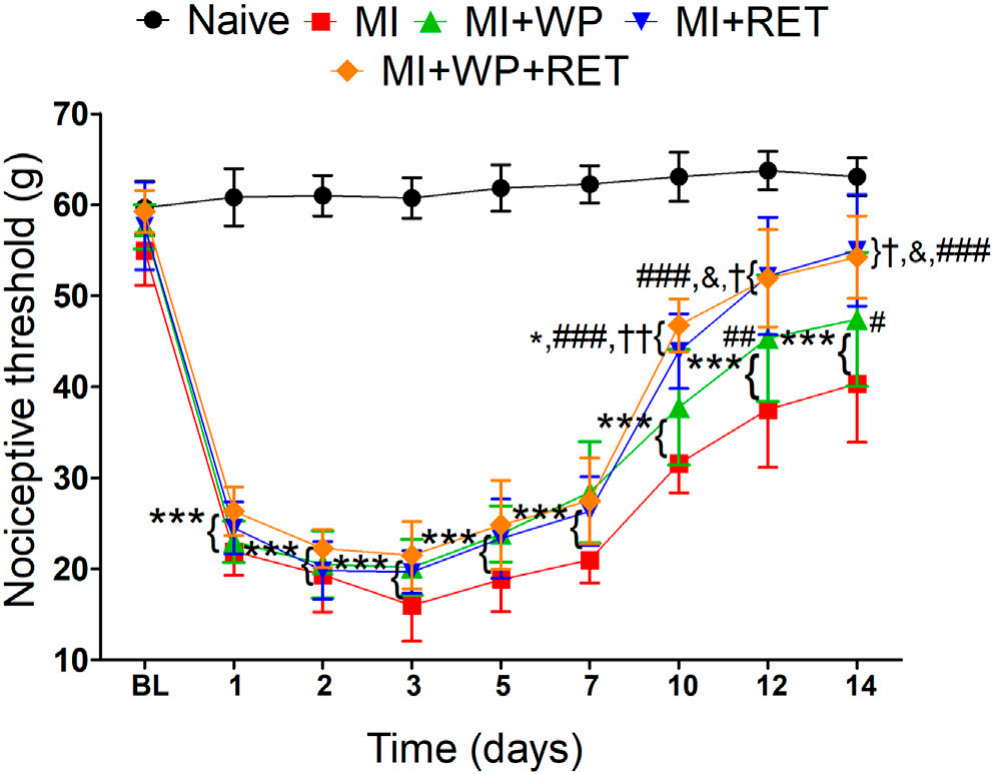
Effect of the resistance exercise training and whey protein supplementation on the muscle pain induced by injury. First, the baseline (BL) nociceptive threshold of each animal was measured and then the muscle injury (MI) was induced. After that, rats were subjected to resistance exercise training (RET) and/or whey protein (WP) supplementation and new measurement of the nociceptive threshold was performed after the 1st and 2nd days and every 2 days until the 14th day. Data are expressed as the mean ± S.E.M. of six animals per group. *p < 0.05 and ***p < 0.001 indicate statistical significance compared to the naïve group; *p < 0.05, **p < 0.01, and ***p < 0.001 indicate statistical significance compared to the MI group; ^&^ *p* < 0.05 indicates statistical significance between MI + RET and MI + WP groups; and *p < 0.05 and **p < 0.01 indicate statistical significance between MI + WP + RET and MI + WP groups. Two-way ANOVA was followed by the Bonferroni *post hoc* test.

Studies have demonstrated that both exercise and whey protein supplementation may reduce muscle pain (Galdino et al., 2014; Jones, 2015; Qin et al., 2019). Thus, in this study, we investigated the effect of both treatments, alone and in combination, on the nociceptive threshold of rats with trauma-induced muscle allodynia. Rats with muscle injury presented a significant reduction [F(4,24) = 13.16; *p* < 0.001] in the nociceptive threshold from day 1 to 14 after injury (Figure 3). However, it can be seen that this response decreased on days 7 [F(4,24) = 13.16; *p* < 0.05], 12 [F(4,24) = 13.16; *p* < 0.01], and 14 [F(4,24) = 13.16; *p* < 0.05] in injured animals that received whey protein supplementation. In addition, rats with muscle injury subjected to resistance exercise training and resistance exercise training plus whey protein supplementation also showed an increase in the nociceptive threshold on days 10, 12, and 14 [F(4,24) = 13.16; *p* < 0.001] after injury in comparison with untreated rats with muscle injury. When comparing the groups that received treatments, the injured animals subjected to both resistance exercise training and whey protein plus resistance exercise training showed a higher nociceptive threshold than those that received only whey protein supplementation on days 10 [F(4,24) = 13.16; *p* < 0.01 for resistance exercise plus whey protein *versus* whey protein], 12, and 14 [F(4,24) = 13.16; *p* < 0.05 for resistance exercise and resistance exercise plus whey protein versus whey protein] after injury (Figure 3). These results demonstrate that resistance exercise training or whey protein supplementation, as well as their combination, was effective in reducing muscle injury–induced allodynia.

Whey protein supplementation or vehicle did not alter the nociceptive threshold in the naïve group (data not shown).

### Improved Gait During Muscle Injury due to Resistance Exercise Training and Whey Protein Supplementation

**FIGURE 4:**
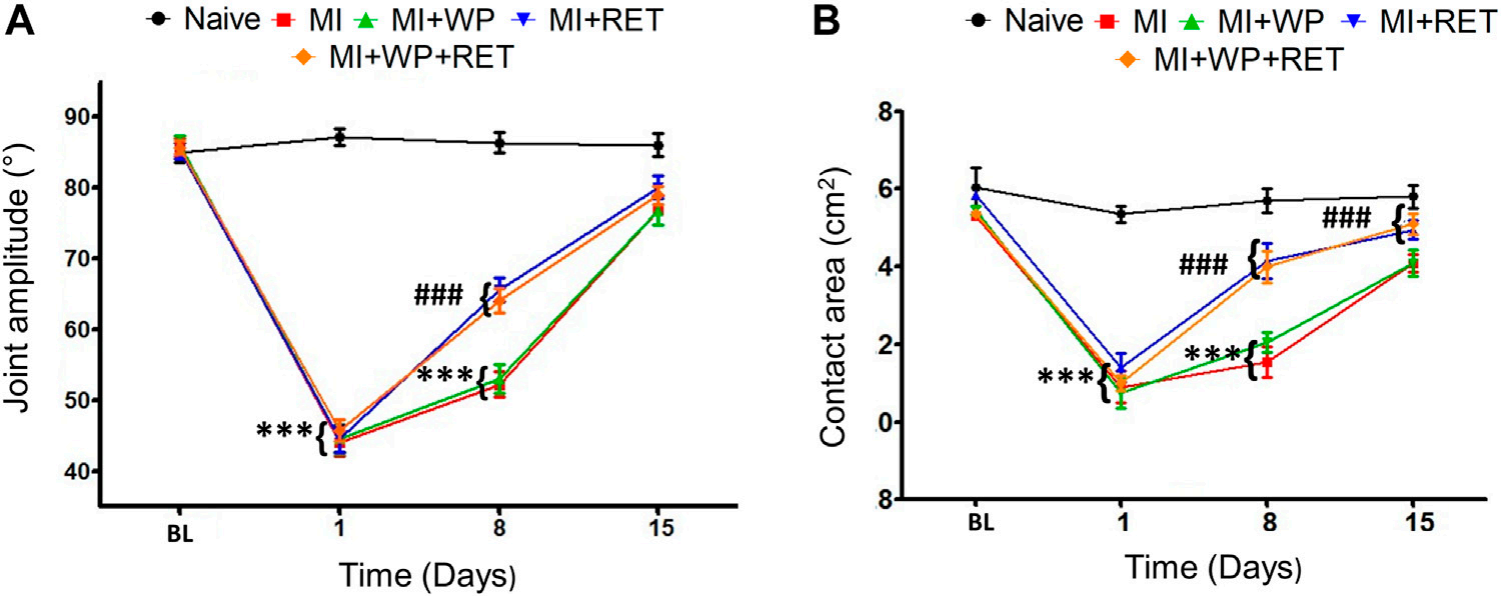
Effect of the resistance exercise training (RET) and whey protein (WP) supplementation on the gait during muscle injury. First, the baseline (BL) of joint amplitude **(A)** and contact area **(B)** of the gait of each animal were evaluated, and a new evaluation was performed on the 8th and 15th days of muscle injury (MI). Data are expressed as the mean ± S.E.M. of six animals per group. ***p < 0.001 indicates statistical significance compared to the naïve group; ***p < 0.001 indicates statistical significance compared to the MI group. Two-way ANOVA was followed by the Bonferroni *post hoc* test.

Muscle injury generally affects gait performance. Therefore, we evaluated whether the muscle injury model proposed herein interferes with the gait of animals. To investigate the effect of muscle injury, as well as the effects of treatment on the animal’s gait, we analyzed the joint amplitude of motion and contact area of the paw. Our results demonstrated that the group with muscle injury presented a decreased joint amplitude [F(4,24) = 13.16; *p* < 0.001] 1 and 8 days after injury compared to the naïve group (Figure 4A). Resistance exercise training and resistance exercise training plus whey protein supplementation increased the joint amplitude on the 8th day [F(4,24) = 13.16; *p* < 0.001] compared to muscle injury group. Regarding the contact area, Figure 4B shows a significant reduction [F(4,24) = 13.16; *p* < 0.001] after the 1st and 8th days of muscle injury. This reduced response was attributed to resistance exercise training and resistance exercise training plus whey protein supplementation treatment [F(4,24) = 13.16; *p* < 0.001] on the 8th day of injury (Figure 4B). Treatment with whey protein supplementation did not reduce the contact area in injured animals. Together, these results reveal that resistance exercise training and resistance exercise training plus whey protein supplementation can improve the gait during muscle injury. Whey protein supplementation or vehicle did not alter the gait in the naïve group (data not shown).

### Effect of Resistance Exercise Training and Whey Protein Supplementation on the Inflammatory Response in Injured Muscle

**FIGURE 5:**
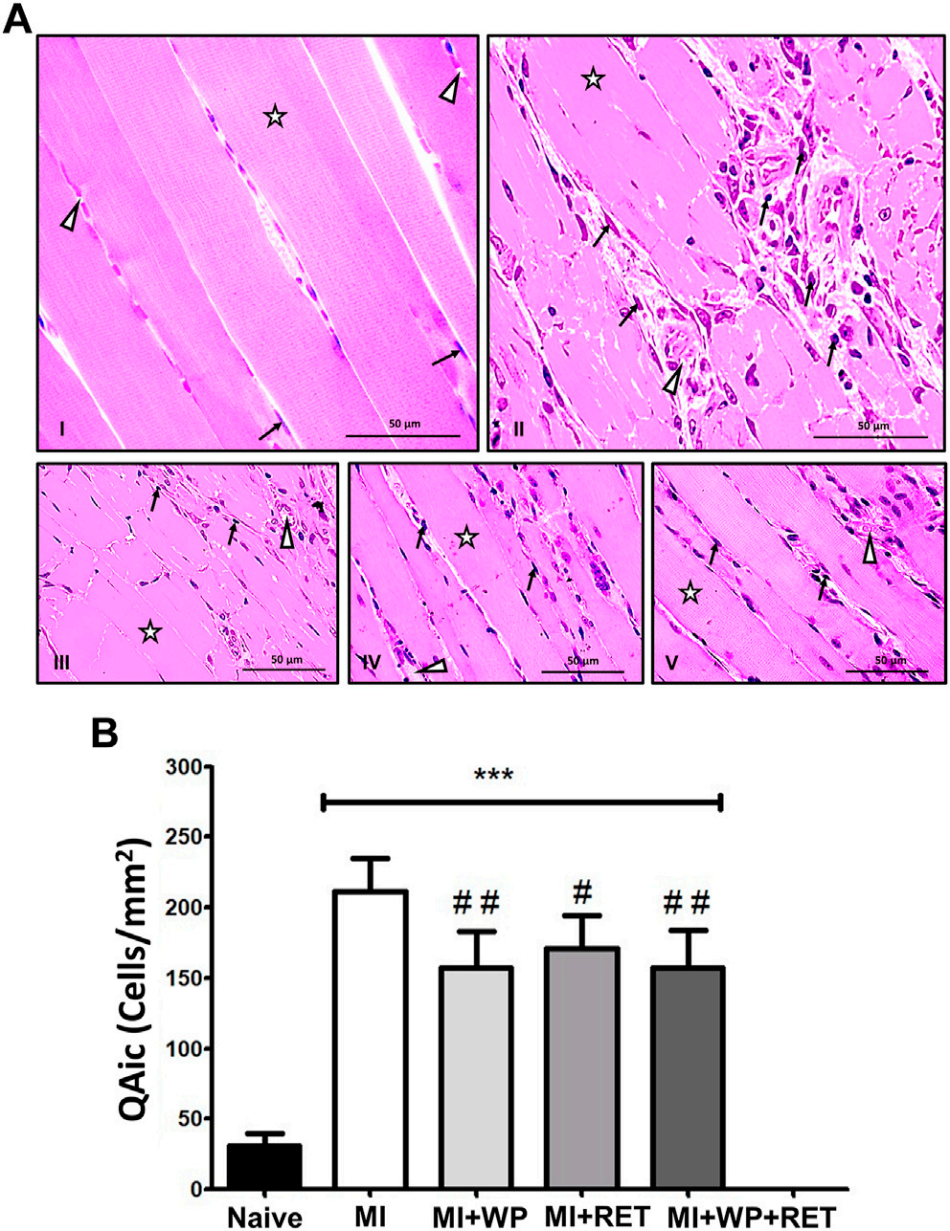
Effect of the resistance exercise training (RET) and whey protein supplementation (WP) on the gastrocnemius muscle interstitial cellularity in the gastrocnemius muscle after the 2nd week of muscle injury (MI). **(A)** Representative histological aspect of the gastrocnemius muscle from control and treated animals (star: skeletal myocyte, arrow: nuclei of interstitial cells, arrowhead: blood vessel). Groups: I = naive, II = MI, III = MI underwent WP, IV = MI underwent RET, and V = MI underwent WP plus RET. **(B)** Number density of interstitial cells (QAic) in the gastrocnemius muscles of rats underwent RET, WP, and RET plus WP. Data are expressed as the mean ± S.E.M. of six animals per group. ***p < 0.001 indicates the statistical significance compared to the naïve group; ^#^ *p* < 0.05 and ^##^ *p* < 0.01 indicate statistical significance compared to the MI group. One-way ANOVA was followed by the Bonferroni *post hoc* test.

Muscle injury typically initiates a rapid and sequential invasion of inflammatory cell populations in the muscle (Gibbins, 2018; Treede, 2018). This invasion can persist for days or weeks while muscle repair, regeneration, and growth occur (Laumonier and Menetrey, 2016). Thus, 15 days after muscle injury, histological analysis of the right gastrocnemius muscle tissue was performed in order to evaluate the density of interstitial cells, as well as the level of injury and the effect of treatment. The number of interstitial cells used as an indirect marker of muscle inflammatory damage (Novaes et al., 2017) increased significantly [F(4,24) = 13.16; *p* < 0.001] in injured animals with and without treatment compared to the naïve group (Figure 5). However, in the group with muscle injury subjected to resistance exercise training [F(4,24) = 13.16; *p* < 0.05], whey protein supplementation [F(4,24) = 13.16; *p* < 0.01], or resistance exercise training plus whey protein supplementation [F(4,24) = 13.16; *p* < 0.01], this increase was smaller (Figure 5). There was no statistical difference among the treated groups.

**FIGURE 6:**
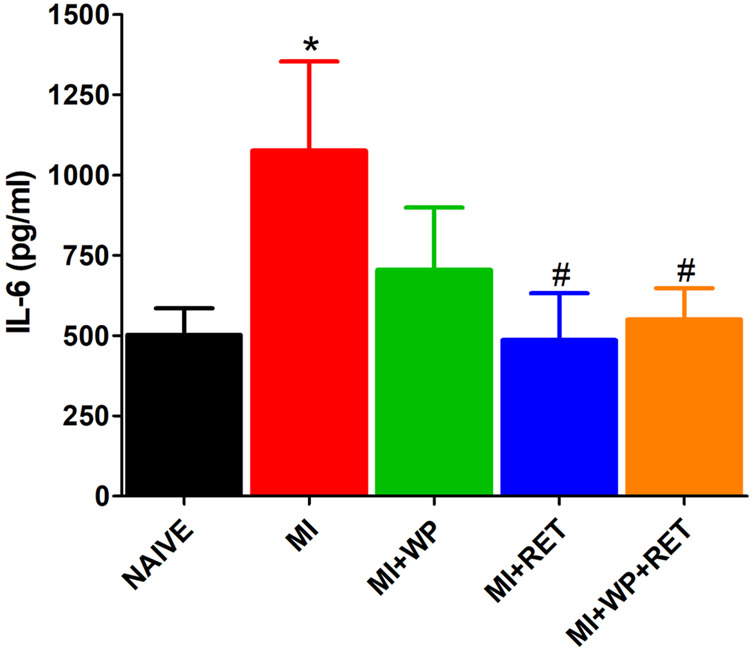
Effect of resistance exercise training (RET) and/or whey protein supplementation (WP) on the pro-inflammatory cytokine IL-6 muscle levels. Data are expressed as a mean ± S.E.M of 5–6 animals per group. *p < 0.05 indicates a statistical difference compared to the naïve group; *p < 0.05 indicates statistical significance compared to the muscle injury (MI) group. One-way ANOVA followed by the Bonferroni test.

In addition, the present study also demonstrated that the pro-inflammatory cytokine IL-6 levels significantly (*p* < 0.05) increased in the right gastrocnemius muscle 15 days after injury (Figure 6), and was significantly (*p* < 0.05) reduced by resistance exercise training alone or when combined with whey protein supplementation. In view of these findings, we believe that the proposed treatments may attenuate the inflammatory process in the injured muscle.

### Participation of the Spinal Glial Cells in Trauma-Induced Muscle Injury

**FIGURE 7:**
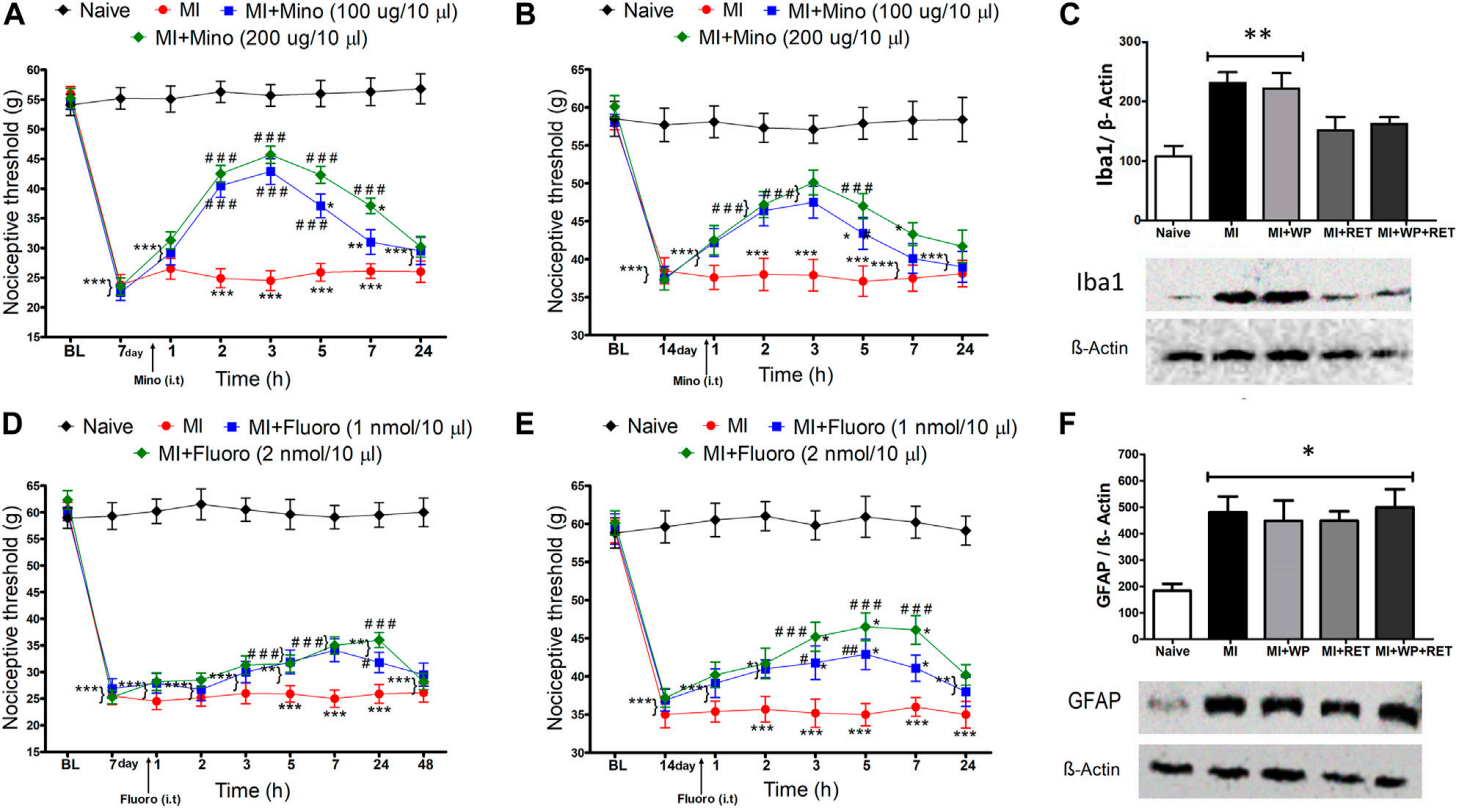
Spinal involvement of the glial cells in the muscle injury–induced nociception. On the 7th or 14th day after the injury, minocycline [Mino, **(A,B)**] or fluorocitrate [Fluoro, **(D,E)**] was intrathecally administered, and new measurements of the nociceptive threshold were performed after 1, 2, 3, 5, 7, 24, or 48 h. After the 2nd week of the resistance exercise training (RET) and/or whey protein (WP) supplementation, the Iba1 **(C)** and GFAP **(F)** protein-level expression in the spinal cord was analyzed in rats with and without muscle injury (MI). Data are expressed as the mean ± S.E.M. of 4–6 animals per group. *p < 0.05, **p < 0.001, and ***p < 0.001 indicate statistical significance compared to the naïve group; ***p < 0.05, ^##^ *p* < 0.01, and ^###^ *p* < 0.001 indicate statistical significance compared to the MI group. One (for Western blot assay) and two-way (for pharmacological assays) ANOVA followed by the Bonferroni *post hoc* test.

Spinal glial cells are involved in the initiation and maintenance of pain, including muscle pain (Ji and Berta, 2013). In this context, the current study evaluated the role of spinal glial cells, such as microglia and astrocytes, in trauma-induced muscle injury. First, we employed the inhibitors minocycline and fluorocitrate, respectively. Minocycline injection significantly reduced mechanical allodynia [F(4,24) = 18.42; *p* < 0.001], which lasted 5 h when using a 100-µg dose on days 7 and 14 after injury, and 7 and 5 h when using a 200-µg dose on days 7 and 14, respectively (Figures 7A,B).

In addition, the intrathecal administration of fluorocitrate using 1- and 2-mmol doses also promoted reduction in mechanical allodynia [F(4,24) = 3.51; *p* < 0.001] on days 7 and 14 after injury, which lasted 24 and 7 h, respectively (Figures 7D,E). Pretreatment with vehicle or drugs (minocycline or fluorocitrate) in non-injured rats did not alter the nociceptive threshold (data not shown). Indeed, this demonstrates that spinal glial cells, mainly microglia, participate in trauma-induced muscle injury.

### Inhibition of Spinal Microglia Protein Levels Promoted by Resistance Exercise Training and Whey Protein Supplementation Treatments

After verifying the participation of glial cells in trauma-induced muscle injury using pharmacological and behavioral experiments, the next step of the study was to evaluate the effect of resistance exercise training and whey protein supplementation on spinal Iba1 (a microglia marker) and GFAP (an astrocyte marker) protein expressions. It was found that muscle injury induced an increase in the Iba1 protein expression [F(4,15) = 5.22; *p* < 0.01] compared to the naïve animal group (Figure 7C), reinforcing our pharmacological results. Moreover, this Iba1 increase was not verified in injured animals submitted to resistance exercise training or resistance exercise training plus whey protein supplementation (Figure 7C). Even though the GFAP protein expression also increased in injured rats [F(4,17) = 16.54; *p* < 0.05] compared to the naïve group, no treatment was responsible for altering this response (Figure 7F). In view of the previous results, we suggest that spinal astrocytes and microglia may be activated in the proposed model of trauma-induced muscle injury. In addition, resistance exercise training alone or combined with whey protein supplementation inhibited only microglia activation.

## Discussion

The current study showed that resistance exercise training and/or whey protein supplementation reduced mechanical allodynia after muscle injury. Additionally, it was demonstrated herein that spinal astrocytes and microglia are involved in the proposed model of trauma-induced muscle injury, and that resistance exercise training alone or combined with whey protein supplementation prevented spinal microglial activation.

Although the nociceptive threshold was not measured in the muscle, the evaluation of mechanical allodynia in the ipsilateral paw of the muscle injury may indirectly suggest a response originating from the trauma-induced injury. Other studies have also found this effect after the administration of inflammatory or allogeneic substances to minimize gastrocnemius muscle pain (Sluka et al., 2001; Radhakrishnan et al., 2003; Radhakrishnan et al., 2004; Dos Santos et al., 2019). A possible explanation would be the development of a secondary hyperalgesia (Kehl et al., 2000); in this case, allodynia in response to a central sensitization (Willis and Coggeshall, 1991), which may be induced by spinal microglia activation.

After validating the mechanical allodynia induced by the muscle injury model, we verified whether the resistance exercise training protocol provoked an overload and physical effort to the animals. To this end, we evaluated the blood lactate level, which has been used as a parameter for the metabolic response provided by different resistance training protocols (Buitrago et al., 2012; Buitrago et al., 2013). The results pointed to elevated blood lactate levels in all rats subjected to resistance exercise training, showing that this increase was higher as the load increased. These data evidenced that the employed resistance exercise training protocol promoted metabolic and muscle overload.

Moreover, resistance exercise training was found to relieve mechanical allodynia in rats that underwent trauma-induced muscle injury. Some studies have reported the mechanisms of antinociception using a weight-lifting exercise model similar to the one used here (Galdino et al., 2010; Galdino et al., 2014). However, these studies did not use a pain model. Furthermore, just few studies have demonstrated the antinociceptive effect of resistance exercise on an injury model. In these studies, the rats performed resistance exercises in water with a load attached to their body. The authors found a control of allodynia induced by formalin injected into the knee joint or calcaneal tendon trauma (Antunes et al., 2012; Bertolini et al., 2012). Clinical studies have also demonstrated that resistance exercise relieves pain in fibromyalgia patients (Jones, 2015). In addition, some other studies have shown that resistance exercise reduces the levels of pro-inflammatory cytokines (Soares and de Sousa, 2013; Abd El-Kader and Al-Shreef, 2018), which was verified herein by the IL-6 reduction in the injured muscle. These cytokines may modulate pain in several ways, such as through the activation of their receptors on the primary afferent fibers in the muscle as well as in the postsynaptic nerve terminal, which can lead to rapid changes in neuronal excitability (Gonçalves Dos Santos et al., 2020). In agreement with the findings previously presented, we suggest that the antinociceptive effect due to exercise resistance training may be through the activation of the descending pain modulation pathway and/or the inhibition of inflammatory mediators.

The present study also demonstrated that whey protein supplementation reduced mechanical allodynia induced by muscle injury. However, this effect was lower than the effect observed when resistance exercise training or resistance exercise training combined with whey protein supplementation was employed. One hypothesis for the antinociceptive effect of whey protein supplementation is also based on the ability to reduce pro-inflammatory cytokines. Although there was a reduction of IL-6 in the injured muscle, such decrease was found only in the group that underwent resistance exercise training combined with whey protein supplementation. However, it has already been reported that whey protein supplementation reduces pro-inflammatory cytokine IL-6 and tumor necrosis factor alpha (TNF-α) levels in patients with ischemic stroke, chronic obstructive pulmonary disease, and hypertension (Lee et al., 2010; Sugawara et al., 2012; Hashemilar et al., 2020). In addition, an *in vitro* study found that the physiological concentrations of dairy whey protein normalize the TNF-α–induced pro-inflammatory gene expression in human endothelial cells (Da Silva et al., 2017). Furthermore, leucine, one of the main amino acids present in whey protein, was found to alleviate muscle damage induced by eccentric contraction. This effect was associated with the reduction of pro-inflammatory IL-6 levels in the muscle (Kato et al., 2016). Leucine may activate the mammalian target of rapamycin (mTOR) signaling (Gran and Cameron-Smith, 2011), a pathway identified as a critical regulator of immune function. In addition, mTOR overexpression suppresses IL-6 secretion from cardiomyocytes exposed to lipopolysaccharides (Song et al., 2010), as well as inflammation in the heart after ischemia/reperfusion injury (Aoyagi et al., 2012). Nevertheless, most of the aforementioned studies did not investigate the effect of whey protein on pro-inflammatory cytokines in trauma-injured muscles. Therefore, we suggest that future studies be carried out to better unravel this mechanism.

When evaluating the combined effect of resistance exercise training and whey protein supplementation, we verified no potentiation of antinociception. Hence, the antinociception effect was likely caused by resistance exercise training. Additionally, the resistance exercise training protocol alone was more effective than the whey protein supplementation treatment. We can then conclude that both treatments are effective in relieving mechanical allodynia in rats with trauma-induced muscle injury, indicating the resistance exercise training showed better results.

The gait analysis was important to evaluate the animal’s motor function during inflammation and in pain situations, such as muscle injury (Xu et al., 2019). In the current study, we found that both resistance exercise training and resistance exercise training plus whey protein supplementation improved the gait parameters in rats with muscle injury. Most studies reporting an improvement in gait due to resistance exercise evaluated this response in patients with neurological or neurodegenerative disorders (Gutierrez et al., 2005; Mehta et al., 2012; Corcos et al., 2013). The present study was the first to evaluate the effect of resistance exercise training on the gait of rats with muscle injury. Brown et al. (2003) conducted a study with rats without injury and also reported an improvement in gait stride and step length. Therefore, we hypothesize that the improvement in gait found herein may be due to the analgesic and anti-inflammatory effect of the treatments used.

Supporting this previous hypothesis, our histological results demonstrated that resistance exercise training alone or combined with whey protein supplementation reduced the pathological microstructural remodeling response induced by muscle injury. Although interstitial cellularity is used as a marker of muscle microstructural inflammatory damage (Novaes et al., 2017; Souza-Silva et al., 2020), the cell types involved in muscle inflammation and repair have not been investigated. Thus, a potential relationship between interstitial muscle cell phenotype and pain pathophysiology should be investigated in future studies. However, a previous study already reported that whey protein supplementation controls the pro-inflammatory response increase in the muscle, possibly resulting from mTOR pathway activation (Kato et al., 2016). The reduction of pathological microstructural remodeling observed in our study may be potentially linked to the immunomodulatory effect, which demonstrated a significant decrease of IL-6 levels in the injured muscle after resistance exercise training combined or not with whey protein supplementation compared to non-treated animals. Novaes et al. (2017) also found a significant attenuation reduction in the skeletal muscle pathological remodeling induced by *Trypanosoma cruzi* infection in rats after exercise training. This response was associated with the pro-inflammatory cytokine TNF-α and pro-inflammatory chemokines CCL-2/MCP-1 and CX3CL1. These results indicate that physical training may be a treatment strategy for inflammatory control, and consequently for pathological microstructural remodeling of the injured muscle.

The present study also showed that spinal glial cells are involved in trauma-induced muscle injury, and that resistance exercise training alone or combined with whey protein supplementation prevented the spinal microglial activation. Although there is a study in the literature demonstrating this effect through exercise (Dos Santos et al., 2019), it did not use resistance exercise; however, they did not use resistance exercise. It is known that during exercise, several neurotransmitters are released, that is, endocannabinoids, which may act on specific receptors in the spinal microglia, inhibiting their activation (Hashemilar et al., 2020). Furthermore, whey protein supplementation may be associated with mTOR activation in the microglia, which may inhibit these cells (Xie et al., 2014).

In summary, this study indicates that resistance exercise training and whey protein supplementation may be efficient treatment against mechanical allodynia after trauma-induced muscle injury. Furthermore, when combined, these treatments may reduce the activation of spinal glial cells during muscle injury. These findings could be important for future studies that investigate the control of pain resulting from muscle injury in patients and athletes.

## Data Availability

The original contributions presented in the study are included in the article/Supplementary Material; further inquiries can be directed to the corresponding author
